# Bladder Trigone as a Sensory Hub: A Narrative Review

**DOI:** 10.7759/cureus.94951

**Published:** 2025-10-19

**Authors:** Takuya Sadahira, Yuki Maruyama, Yosuke Mitsui, Takanori Sekito, Tomofumi Watanabe, Masami Watanabe

**Affiliations:** 1 Department of Urology, Okayama University Graduate School of Medicine, Dentistry and Pharmaceutical Sciences, Okayama, JPN; 2 Center for Innovative Clinical Medicine, Okayama University Hospital, Okayama, JPN

**Keywords:** bladder trigone, botulinum toxin, lower urinary tract symptoms, sensory afferents, varicosities

## Abstract

The bladder trigone is an anatomically and functionally distinct region within the lower urinary tract (LUT), characterized by a dense network of afferent sensory fibers, specialized urothelial interactions, and prominent mechanotransduction mechanisms. Its intricate neuroarchitecture enables precise detection of bladder filling and coordination of micturition, whereas dysregulation of these pathways contributes to lower urinary tract symptoms (LUTS), including urgency, frequency, and bladder pain. Despite its recognized clinical relevance, the structural and functional basis of trigonal sensory signaling - and its role - remain incompletely understood.

This review synthesizes current evidence on trigonal afferent organization, integrating data from anatomical mapping, receptor profiling, electrophysiological characterization, and translational research. Seminal anatomical observations are combined with recent advances in mechanotransduction and purinergic, peptidergic, and transient receptor potential (TRP) signaling to provide a comprehensive perspective. The trigone exhibits three principal afferent classes: (1) intraepithelial fibers penetrating umbrella cells, marked by P2X purinoceptor 3 (P2X3), transient receptor potential vanilloid 1 (TRPV1), calcitonin gene-related peptide (CGRP), and substance P (SP); (2) subepithelial plexuses surrounding microvasculature, enriched in vasoactive neuropeptides and exhibiting plastic hypertrophy in overactive bladder (OAB) and interstitial cystitis/bladder pain syndrome (IC/BPS); and (3) encapsulated corpuscular endings at the lamina propria-detrusor junction, expressing PIEZO1/2 and acid-sensing ion channels (ASICs) for rapid adaptation. In trigeminal dorsal root ganglion (DRG) neurons, high expression of PIEZO2, P2RX3, and voltage-gated sodium channel, type 1.8 (Nav1.8) was observed, revealing their role as the foundation for multisensory information processing. Functional assays highlight distinct mechanotransductive and chemosensory pathways, with aging, inflammation, and neurotrophic factors driving afferent plasticity underlying abnormal bladder sensation, such as urgency, frequency, and pain. Early clinical trials of P2X3 antagonists and intravesical TRPV1 inhibitors demonstrate promising symptomatic benefits. Collectively, evidence positions the bladder trigone as a critical sensory hub where neuronal, urothelial, and immune signals converge to regulate bladder sensation. Understanding its molecular and structural specialization may inform the development of region-specific neuromodulatory therapies targeting sensory urgency and afferent-driven bladder dysfunction.

## Introduction and background

The lower urinary tract (LUT) meticulously orchestrates a delicate balance between the critical functions of urine storage and controlled, voluntary voiding [1]. This remarkable physiological feat is achieved through a sophisticated interplay of coordinated detrusor muscle contraction and intricate sensory feedback mechanisms [2]. The bladder trigone is the center of this essential sensory feedback loop and is a smooth, triangular region bounded by the two ureteral orifices superiorly and the internal urethral meatus inferiorly. While historically dismissed as a passive region primarily serving to guide urine flow into the bladder neck, emerging evidence reveals that the trigone is a highly specialized and functionally dynamic zone of the LUT [3].

Anatomical and neurophysiological studies have demonstrated that the trigone possesses a distinct anatomical structure, different from the bladder wall and dome, is innervated by a rich network of afferent fibers, and exhibits a high sensitivity to stretch and chemical stimuli [4]. The trigone possesses a notably stiff extracellular matrix (ECM), richly endowed with collagen types I and III, and conspicuously lacks the prominent urothelial rugae typically found in other bladder regions [5]. This inherent lack of compliance within the trigone means that mechanical forces generated during bladder filling are not dissipated across distensible folds, but are instead concentrated directly onto its embedded sensory nerve endings. This focused mechanical stimulation is crucial for the early and precise detection of bladder fullness.

The foundational understanding of the trigone's sensory importance stems from pioneering histological investigations. Seminal studies meticulously documented the existence of dense intraepithelial and subepithelial nerve networks within the trigone, laying the groundwork for subsequent research [6,7]. In recent decades, technological advancements have dramatically enhanced our ability to investigate complex neural networks. Methods such as whole-mount tissue clearing, 3D confocal microscopy, and optogenetics have revolutionized the field by offering unparalleled spatial and temporal resolution for mapping sensory innervation. These cutting-edge techniques have not only corroborated early findings, but have also substantially expanded our understanding - revealing that fibers derived from the dorsal root ganglion (DRG) invade the trigone during embryonic development and maintain a strong sensory presence into adulthood. Furthermore, the advent of optogenetics and chemogenetics has equipped researchers with powerful tools to probe the causal roles of specific neuronal populations in the regulation of micturition [8].

This review integrates the most recent anatomical, molecular, and clinical evidence to highlight the bladder trigone’s unique afferent architecture and its implications in abnormal bladder sensation and afferent-driven dysfunction. We focus on the following key dimensions: the anatomical and cellular basis of trigonal afferents, molecular transduction pathways critical for sensory encoding, and the translational relevance in disease mechanisms and emerging therapies.

## Review

Methods

Eligibility Criteria

A search for full-text articles in two large bibliographic databases, PubMed and Google Scholar, was performed for articles that describe the anatomical and cellular basis of trigonal afferents, molecular transduction pathways critical for sensory encoding, and translational relevance in disease mechanisms and emerging therapies. Articles not written in English and those for which the full text was unavailable were excluded.

Study Selection and Data Collection Process

The literature search was conducted on June 24, 2025. Our search used the following terms or keywords: “Bladder trigone,” “Sensory afferents,” “Varicosities,” “Lower urinary tract syndrome,” and “Botulinum toxin.” First, titles were screened for articles of interest. The search engine Google was also used, yielding additional articles. Snowballing was employed when possible. Table 1 summarizes our methodology in this article.

**Table 1:** Research strategy summary

Items	Specification
Date of search	June 24, 2025
Databases and other sources searched	PubMed, Google Scholar. Snowball of articles was also included
Search terms used	Bladder trigone, Sensory afferents, Varicosities, Lower urinary tracts syndrome, Botulinum toxin
Timeframe	Up to 2025
Exclusion criteria	Articles not written in the English language and those for which the full text was unavailable were excluded
Selection process	The first author conducted all searches, and any doubts in the selection process were evaluated by co-authors 2 and 6

Gross and histological anatomy of the bladder trigone

From a macroscopic perspective, the bladder trigone presents as a strikingly smooth-walled, non-distensible triangular area, serving as a critical anatomical gateway that connects the upper urinary tract (via the ureteral orifices) to the LUT's exit point (the internal urethral meatus) [9]. Morphometric analyses have provided quantitative insights into its structural distinctiveness, revealing that the trigonal wall thickness typically ranges from 0.46 to 1.58 mm, notably thinner compared to the bladder dome, which can measure between 0.98 and 3.07 mm in thickness [10]. This disparity in thickness contributes to the trigone's relative rigidity and limited compliance, consistent with anatomical reviews noting its smooth, unruffled mucosa and shape preservation regardless of bladder filling [11].

At the microscopic level, the trigone's urothelium is characterized as stratified transitional epithelium, much like the rest of the bladder lining. However, subtle but significant differences exist. Although the basic architecture - comprising basal, intermediate, and superficial (or surface) cell layers - is common throughout the bladder urothelium, it has been reported that the trigonal urothelium consists of approximately three cell layers, making it thinner than the urothelium in other regions of the bladder (three to six layers) [12]. The umbrella cell layer, which forms the outermost protective barrier, has been observed to be thinner in the trigone. Paradoxically, this thinner layer exhibits a higher expression of tight junction proteins such as zonula occludens-1 (ZO-1) and occludin, along with regionally elevated claudin-1 and claudin-4 transcripts [13]. These findings align with recent mucosal immunology work suggesting that the trigone possesses a more robust and impermeable barrier than the dome, potentially protecting underlying neural elements from urinary solute exposure [14].

Morphometric and ultrastructural analyses have highlighted several distinctive sensory features of the bladder trigone. First, intraepithelial sensory nerve endings are abundant within the urothelial layer, extending between umbrella cells and terminating close to the luminal surface, as demonstrated by fine structural and immunohistochemical studies [6,15,16]. Second, the urothelium of the trigone is relatively thin, usually consisting of three to four cell layers, whereas the bladder dome often displays up to six layers [12]. This thinner urothelium facilitates close apposition of afferent terminals to the luminal environment. Third, suburothelial nerve plexuses with necklace-like varicosities are consistently localized within approximately 0.5 mm beneath the urothelial basement membrane [6,15-17]. These varicosities contain dense-core and clear vesicles filled with neuropeptides and classical neurotransmitters, forming the structural substrate for amplified sensory signaling. Finally, comparative neuroanatomical mapping confirms that the trigone harbors the highest density of sensory afferents in the bladder, exceeding that of the dome or lateral walls [7,17].

Collectively, these findings establish the trigone as a structurally unique and sensory-dense region, optimized for detecting bladder filling and mediating abnormal bladder sensation in pathological states (summarized in Table 2). Beneath the urothelium lies the lamina propria, a highly specialized connective tissue layer composed of ECM, interstitial cells, nerve endings, and a rich vascular-lymphatic supply [18]. In the trigone, the lamina propria contains a dense suburothelial capillary network situated within a few micrometers of the urothelial basement membrane, and these vessels, together with an abundant lymphatic system, contribute to a specialized “neurovascular-lymphatic niche” that supports metabolic exchange, immune surveillance, and afferent nerve function [19]. This intricate microenvironment is well-positioned to provide metabolic support and waste clearance for the sensory nerve fibers it houses [19].

**Table 2:** Comparison between the bladder trigone and other regions

	Bladder trigone	Other regions (ex. bladder dome)
Morphology	Smooth	Ruffled
Compliance	Lacking	High
Wall thickness	Thin (0.46-1.58 mm)	Thick (0.98-3.07 mm)
Urothelium	Transitional epithelium	Transitional epithelium
Urothelial cell layer	Thin (3-4 layers)	Thick (3-6 layers)
Umbrella cell layer	Thin	Thick
Tight junction protein expression in the umbrella cell layer	High	Relatively low
Sensory afferents density	Highest density	Relatively low
Suburothelial afferents	High	Relatively low

Immunohistochemical and three-dimensional mapping studies in humans have elucidated a rich innervation of the trigone, extending from extrinsic nerve trunks to the urothelial layer [7]. Quantitative analysis in aging bladders reveals a reduction in calcitonin gene-related peptide (CGRP)-positive sensory innervation within the trigone, underscoring the functional significance of trigonal afferents in sensory processing [20]. Electron microscopy of the human bladder lamina propria, including trigonal specimens, has demonstrated unmyelinated axonal varicosities consistent with C-fiber terminals, often lying in close apposition to urothelial and suburothelial structures [17]. These varicosities display two distinct vesicle populations: large dense-core vesicles (median diameter ~100-120 nm) and smaller clear vesicles (~40-50 nm), indicating the co-storage of neuropeptides such as substance P (SP) and classical neurotransmitters including acetylcholine (ACh) or adenosine triphosphate (ATP) [15]. Although rare, specialized encapsulated sensory endings have been observed; these are composed of concentric Schwann cell lamellae and are hypothesized to serve a mechanosensory function. Recent molecular profiling has revealed the presence of mechanotransduction-associated proteins such as PIEZO2 and synaptophysin in suburothelial afferent terminals, reinforcing their role in detecting mechanical stimuli and modulating LUT reflexes [21].

This detailed anatomical characterization underscores the trigone's rich and diverse innervation, setting the stage for its complex sensory capabilities.

Afferent architecture of the trigone

The trigone contains a dense population of afferent nerve fibers, including Aδ and C fibers, that convey mechanical, chemical, and inflammatory stimuli to the spinal cord and higher centers [1]. These afferents arise primarily from the DRG at the lumbosacral level (L6-S1 in rodents; S2-S4 in humans) and project to the superficial dorsal horn and sacral parasympathetic nucleus [22]. Compared with the bladder dome, the trigone has a higher density of suburothelial afferents, and many fibers appear to be closely associated with capillaries, myofibroblasts, and interstitial cells - an arrangement consistent with potential neurovascular and neuromodulatory interactions. Three principal classes of afferents have been identified in the trigonal region: (i) mechanosensitive low-threshold Aδ fibers, (ii) polymodal C fibers, and (iii) silent afferents.

Mechanosensitive Low-Threshold Aδ Fibers

These myelinated fibers respond to bladder filling and stretch, and are believed to encode bladder fullness. PIEZO2, a mechanosensitive ion channel critical for encoding stretch in visceral tissues, has recently been suggested to be localized in these Aδ fibers in the human bladder trigone, supporting their role in conscious bladder filling sensation [21].

Polymodal C Fibers

These unmyelinated fibers are less responsive to low-threshold mechanical stimuli and are primarily responsible for transmitting slow, diffuse sensations, but they are activated by noxious stimuli such as ATP, low pH, and inflammatory mediators. They express high levels of transient receptor potential vanilloid 1 (TRPV1) and P2X purinoceptor 3 (P2X3) receptors, and are significantly involved in pain sensation and urgency, particularly in pathological states such as interstitial cystitis/bladder pain syndrome (IC/BPS) and overactive bladder (OAB) [23].

Silent Afferents

These fibers are normally unresponsive to physiological stimuli, but become active in response to inflammation or tissue damage. Their sensitization involves upregulation of neurotrophic factors, such as nerve growth factor (NGF) and brain-derived neurotrophic factor (BDNF), as well as upregulation of chemosensitive channels, including transient receptor potential ankyrin 1 (TRPA1) and acid-sensing ion channels (ASICs). These afferents are particularly enriched in the trigone during chronic inflammation, contributing to allodynia and hyperalgesia [24].

Recent studies using 3D neuroanatomical imaging have visualized the spatial organization of afferents in the trigone with high resolution. These methods have confirmed that sensory endings often cluster in the suburothelial space and form networks associated with interstitial cells and submucosal vasculature [7]. Importantly, the proximity of trigonal afferents to immune cells and urothelial signaling molecules creates a bidirectional neuroimmune interface. Mast cells, macrophages, and T cells in the trigonal lamina propria can modulate afferent excitability through cytokine and ATP release, a process especially relevant in chronic inflammatory disorders.

Sensory nerve endings of the bladder trigone

The exquisite sensitivity of the bladder trigone reflects a diverse set of specialized sensory nerve endings that, together, detect and transmit mechanical and chemical cues from the LUT. Contemporary work confirms three principal classes with distinct molecular signatures and functions, and highlights region-specific biology of the trigone compared with the dome [25]. 

Firstly, intraepithelial nerve endings are predominantly unmyelinated fine fibers, often comprising C-fibers [26]. Ultrastructural studies of the human bladder have shown that these fibers are located within the urothelium and immediate suburothelial layers, without evidence of myelin. These fibers are extremely thin (generally <1 µm in diameter) and terminate in close proximity to the umbrella cell layer, with some reports indicating that they extend between or into the basal aspect of umbrella cells [27]. A defining characteristic of these fibers is their co-expression of the purinergic receptor P2X3 and the TRPV1 channel, consistent with active urothelial-afferent crosstalk rather than purely passive detection [26,28]. Electrophysiological and genetic studies now show that low-threshold mechanosensitive currents subserving bladder filling depend on PIEZO2 in both urothelial cells and the innervating sensory neurons; conditional PIEZO2 deletion disrupts low-pressure stretch encoding and voiding reflex timing, and humans with PIEZO2 deficiency report markedly impaired bladder-fill sensation [29]. These data situate PIEZO2 as indispensable to the mechanotransduction capabilities of these endings, with complementary reviews outlining PIEZO channel roles in bladder sensory signaling.

Secondly, subepithelial free nerve endings form an extensive, highly arborized plexus immediately beneath the urothelium, with many fibers coursing in close apposition to the dense microvasculature of the lamina propria [16,30]. These afferents are enriched in neuropeptides such as CGRP, SP, and vasoactive intestinal peptide (VIP), which are associated with neurogenic inflammation, nociceptive signaling, and regulation of vascular tone [16]. These neuropeptides are implicated in local neuroinflammation, nociceptive signaling, and modulation of vascular tone. In pathological conditions such as OAB, suburothelial sensory plexuses exhibit structural plasticity, with several studies reporting substantial increases in nerve fiber density - sometimes by several tens of percent compared with control [31]. Such hypertrophied endings frequently show upregulated immunoreactivity for TRPV1 and P2X3 receptors, changes that are thought to enhance afferent sensitivity to chemical and mechanical stimuli, and may contribute to the urgency and frequency symptoms experienced by patients [32].

Thirdly, though comparatively rare, encapsulated corpuscular endings - resembling Pacinian or Ruffini corpuscles - have been described in the deeper lamina propria, often near the detrusor interface [33]. Their concentric lamellar architecture, formed by Schwann cell layers, is adept at detecting dynamic mechanical stimuli such as rapid changes in intravesical pressure. Interestingly, contemporary clinical and large-animal studies show that intravesical desensitization of TRPV1-positive afferents with resiniferatoxin (RTX) is feasible, reduces SP/CGRP immunoreactivity, and modulates afferent-driven bladder activity - consistent with corpuscular and suburothelial targets being pharmacologically accessible [34]. Collectively, the organization and molecular specialization of these nerve endings underscore the trigone’s central role in relaying sensory information to the central nervous system and shaping the perception of bladder fullness, urgency, and pain - while also illuminating mechanotransduction (PIEZO2), purinergic (P2X3), and TRPV1 pathways as clinically actionable nodes.

Molecular markers and transduction pathways in trigonal afferents

Detection and transmission of mechanical and chemical stimuli in the trigonal afferents is mediated by a complex interplay of various molecular markers and sensory receptors expressed on their afferent nerve endings. Suburothelial afferents in the LUT are commonly identified with general neuronal markers (PGP9.5/UCHL1, βIII-tubulin), the myelinated-fiber marker NF200 (Aδ), and peptidergic nociceptor markers CGRP and SP (C-fiber enriched). Functionally decisive receptor/channel markers include the ATP-gated P2X3 receptor on sensory endings, the heat/chemomechanosensitive TRPV1 channel, and the low-threshold mechanotransducer PIEZO2, expressed in urothelial cells and sensory neurons [21,35]. Together, these markers delineate afferent classes and predict responses to mechanical stretch and inflammatory mediators. Evidence for these assignments derives from P2X3-null mouse phenotypes (hyporeflexia), TRPV1-P2X cross-sensitization, and human genetics/physiology implicating PIEZO2 in bladder filling sensation [28]. In addition to these primary transducers, receptors such as ASIC3, TRPA1, and voltage-gated sodium channel, type 1.8 (Nav1.8) channels contribute to afferent excitability in the trigone.

The initial critical step in trigonal mechanosensation is the opening of pannexin-1 (Panx1) channels in the urothelium [36]. During bladder filling, mechanical stretch concentrates strain within the relatively noncompliant trigone, promoting PIEZO2-dependent mechanotransduction and opening of Panx1 channels in the urothelium, with consequent ATP efflux into the suburothelial compartment. The released ATP activates P2X3 receptors on suburothelial Aδ and C-fiber afferent endings, initiating action-potential discharge that encodes bladder fullness [35]. P2X3-deficient mice display bladder hyporeflexia, with reduced voiding frequency and increased capacity, underscoring the receptor’s necessity. The clinical significance of this pathway is underscored by the efficacy of P2X3 antagonists, and promising Phase II clinical trials have shown that these antagonists can decrease urinary frequency by a remarkable 40% in patients with OAB, highlighting P2X3 as a highly attractive therapeutic target [37].

The next step is to gain control of TRPV1 and PIEZO2. TRPV1 activity enhances P2X-mediated afferent responses, providing a potentiating “gain” on purinergic input. In parallel, PIEZO2 in urothelial cells and sensory neurons supplies low-threshold mechanotransduction that couples physiological stretch to transmitter release and spike initiation. TRPV1 and TRPA1, ligand-gated ion channels, are renowned for their polymodal activation, responding to diverse stimuli including heat (typically >43°C), the pungent compound capsaicin (a key ingredient in chili peppers), and a variety of oxidative mediators (e.g., acrolein and hydrogen peroxide). Their activation leads to the influx of cations, depolarizing the afferent fiber and initiating pain signals, thereby contributing to chemical nociception. TRPV1 antagonists, such as capsazepine derivatives, have shown promise in preclinical models by significantly prolonging inter-micturition intervals during rodent cystometry, suggesting their potential to reduce bladder hypersensitivity [38].

The critical role of PIEZO2 in trigonal mechanosensation has been demonstrated in conditional PIEZO2 deletion studies in DRG neurons, where targeted knockout of the gene encoding PIEZO2 resulted in a significant reduction in afferent firing in response to mechanical stimulation [39]. Neuropeptides also play a significant role in modulating trigonal afferent activity and central nervous system responses. Antidromic release of CGRP and SP from activated afferents augments local neurogenic inflammation, further reinforcing excitability in trigonal afferent fields [40]. This central sensitization is evidenced by elevated levels of phosphorylated extracellular signal-regulated kinase (p-ERK) and c-Fos expression in the spinal cord following trigonal stimulation, both of which are molecular markers of neuronal activation and plasticity associated with persistent pain states [41].

Furthermore, classical neurotransmitters also influence trigonal afferent excitability. ACh, a key neurotransmitter in the parasympathetic nervous system, has been shown to modulate afferent thresholds in the trigone. ACh modulates bladder (including trigonal) afferent excitability via muscarinic M2/M3 receptors and nicotinic receptors expressed in the urothelium and in bladder-innervating DRG neurons [42]. This intricate interplay between various molecular markers and sensory receptors underscores the trigone's highly specialized role in detecting and processing diverse sensory information from the bladder, enabling a nuanced and adaptable response to changes in bladder fullness and potential irritants.

Clinical implications of trigonal sensory specialization

The specialization of sensory innervation within the bladder trigone holds profound clinical implications, particularly in understanding and managing lower urinary tract symptoms (LUTS). An abundance of sensory receptors in the trigone provides important feedback long before the bladder reaches its maximum capacity, constituting a fundamental factor that enables early detection of bladder filling. This early warning system allows for timely and appropriate voiding behavior. The trigonal afferent system exhibits pronounced plasticity in response to inflammation, injury, and chronic bladder pathology.

Inflammation within the bladder trigone promotes the release of pro-inflammatory cytokines such as interleukin-6 (IL-6), tumor necrosis factor-alpha (TNF-α), and NGF, which sensitize afferent nerve endings and upregulate their receptor expression [43]. NGF has been shown to induce sprouting of suburothelial afferents, increase TRPV1 and P2X3 expression, and enhance C-fiber excitability [44]. Clinical studies have demonstrated elevated urinary NGF levels in patients with OAB and IC/BPS, supporting its role as a biomarker and therapeutic target [45,46].

In conditions like OAB, the trigone exhibits significant pathological alterations that directly correlate with the severity of symptoms. Evidence for sensory specialization of the bladder trigone in OAB-related phenotypes is partly supported by human trigonal biopsy data. In women with sensory urgency, TRPV1 mRNA in the trigonal mucosa is elevated relative to the bladder body and inversely correlates with the cystometric volume at first sensation [47]. In healthy tissue, TRPV1 itself does not show a consistent regional gradient, but mRNA profiling of cold-cup biopsies demonstrates higher expression of adhesion/tight-junction genes in the trigone, suggesting distinct barrier properties [48]. In patients with OAB/detrusor overactivity, suburothelial sensory fibers exhibit reductions in P2X3 and TRPV1 after intradetrusor botulinum toxin, aligning receptor signaling with symptom improvement [31].

Furthermore, neuroimaging aligns with a model in which early bladder sensations recruit the insula and anterior cingulate cortex (ACC). Studies employing functional magnetic resonance imaging (fMRI) demonstrate robust insula/ACC BOLD (blood oxygenation level-dependent) increases at the first and strong desire to void, and during urgency episodes [49]. While current fMRI methods don’t isolate the trigone per se, combining these central readouts with the trigone-specific molecular data above supports the view that the trigone is a privileged source of early afferent drive to these cortical salience networks.

Patients suffering from IC/BPS, a debilitating chronic pain condition, also exhibit distinct changes within the trigone. In IC/BPS, classic descriptions emphasized mast-cell-rich neuroinflammation; however, contemporary quantitative pathology shows that mast-cell density varies and is not diagnostic on its own [50]. Bladder biopsies demonstrate increased TRPV1-positive suburothelial fibers that correlate with inflammatory severity; augmented purinergic signaling is supported by increased urinary ATP and stretch-evoked ATP release from patient urothelial cells [51]. Urinary NGF and pro-inflammatory cytokines (e.g., IL-6 and TNF-α) are also elevated in subsets of patients [52]. NGF is a neurotrophin known to promote neuronal sprouting and enhance neuronal excitability, thus linking the inflammatory process directly to afferent sensitization and the chronic pain experienced by these individuals.

The aging process also modifies afferent signaling and perception. Animal work shows reduced CGRP-positive afferent area in the trigonal mucosa with aging, and human urothelial studies report TRPV4 up-regulation in older and OAB bladders [20,53]. Clinically, this can blunt fullness sensation and drive compensatory, pre-emptive voiding. Older individuals may void more frequently, not necessarily due to a true increase in urgency, but because their ability to accurately perceive bladder fullness is impaired. Consequently, differentiating sensory decline from detrusor underactivity - a condition characterized by a weak bladder muscle - becomes crucial for appropriate clinical management. This differentiation can be guided by meticulous assessment of early bladder sensation thresholds through urodynamic studies and detailed analysis of bladder diaries. This personalized approach ensures that tailored management strategies, whether focusing on bladder training, pharmacological intervention, or neuromodulation, are implemented effectively to improve patient quality of life.

Therapeutic implications and emerging strategies

Currently, the clinical evidence for the effectiveness of trigone-targeted therapy, such as trigonal injection of botulinum toxin, is still limited. However, the growing understanding of the bladder trigone's intricate sensory mechanisms is enabling the way for innovative diagnostic and therapeutic strategies, aimed at precisely targeting the underlying pathologies of abnormal bladder sensation. A leading concept is intravesical drug delivery, which concentrates therapy at the urothelium-suburothelial interface while limiting systemic exposure.

P2X3 Pathway Modulation

Intravesical inhibition of P2X3-containing receptors has robust preclinical support: in rat models, intravesical P2X3/P2X2/3 antagonism (for example, AF-353) reduces afferent-driven bladder reflex activity and attenuates overactivity, consistent with a urothelial-afferent purinergic mechanism [54,55]. However, as of 2025, there are no published human trials of intravesical P2X3 antagonists; clinical development has instead focused on oral P2X3 antagonists (e.g., eliapixant) in exploratory OAB programs, where class-specific taste adverse events remain a challenge for systemic dosing [37]. These data collectively motivate local (intravesical/retentive) delivery to exploit the mechanism while minimizing systemic effects.

TRP Channel Targeting

TRP channels expressed in trigonal afferents (notably TRPV1 and TRPA1) remain attractive targets. Intravesical RTX - a potent TRPV1 agonist designed to induce long-lasting desensitization - has yielded mixed clinical results in storage symptoms: small open-label series reported that a single dose improved symptoms and capacity for six months, whereas randomized controlled trials did not show superiority to placebo at commonly used concentrations. These findings support ongoing optimization of dose, dwell time, and patient selection [56,57]. Beyond RTX, TRPV1/TRPA1 antagonists (including dual-inhibitor chemotypes) are advancing in pain pipelines; bladder-specific or intravesical applications remain preclinical/conceptual at present [58].

CGRP Pathway Modulation and Botulinum Toxin

CGRP contributes to neurogenic inflammation and central sensitization, but direct intravesical CGRP-targeted therapies have not yet been established clinically for abnormal bladder sensation and urgency. Preclinical research has progressed: in mice inoculated with uropathogenic *Escherichia coli* (UPEC), the CGRP receptor antagonist BIBN4096BS reduced afferent-driven bladder symptoms and prevented the development of pelvic hypersensitivity [59]. In humans, a prospective observational study is underway to track changes in bladder sensory symptoms and bladder/pelvic pain among migraine patients initiating CGRP-targeted therapies (monoclonal antibodies or gepants) [60]. By contrast, injection of botulinum toxin - including protocols that involve the trigone - is supported by mechanistic human and animal data showing reductions of suburothelial TRPV1, P2X3, and CGRP expression accompanied by symptomatic improvement in OAB/BPS cohorts [31,61]. Trigonal injection is a pathophysiologically rational target for afferent modulation; while RCTs and meta-analyses have reported incremental benefits in neurogenic detrusor overactivity (NDO) and in some idiopathic detrusor overactivity (IDO) cohorts, there are also RCTs that do not demonstrate superiority [62,63].

Neuromodulation

Beyond pharmacological approaches, various neuromodulation techniques are being refined to influence trigonal sensory pathways. Percutaneous tibial nerve stimulation (PTNS) is a minimally invasive procedure that stimulates the tibial nerve at the ankle; by modulating sacral afferent pathways that indirectly influence trigonal activity, it has randomized-trial evidence for clinically meaningful improvements in OAB symptoms and has been shown to increase bladder capacity by roughly 25% [64,65]. Sacral neuromodulation (SNM), a more invasive procedure involving implantable devices, has shown variable effects on direct trigonal signals; it remains an established therapy for refractory OAB [66]. In parallel, the development of novel intravesical electrostimulation catheters, specifically designed to target the trigone, represents a promising technological advancement. Early clinical experiences in OAB/underactive bladder (UAB) suggest improvements in frequency and quality of life [67]. These catheters aim to achieve localized afferent desensitization with minimal impact on detrusor contractility, offering a more precise and potentially safer neuromodulatory approach.

Toward Phenotype-Guided “Trigone-Centric” Precision Care

The next wave of personalization will likely integrate: (i) advanced urodynamic phenotyping (e.g., ambulatory or catheter-free sensors, pressure-electromyography (EMG)/video integration) to resolve early sensation thresholds and compliance with higher fidelity [68]; (ii) urinary biomarker panels (NGF, BDNF, ATP, and others) that associate with OAB severity and treatment response, while acknowledging heterogeneity and the need for standardization [69]; and (iii) molecular profiling - currently research-stage - including single-cell/single-nucleus RNA-seq of bladder tissues and emerging urine-derived cell single-cell RNA sequencing (scRNA-seq), which may ultimately offer non-invasive readouts of urothelial/afferent states [70]. By integrating data from these diverse sources, clinicians will be able to develop phenotype-guided therapies, ensuring that each patient receives the most appropriate and effective intervention based on their unique trigonal sensory profile. These advancements herald a new era of precision medicine in urology, promising more effective and targeted treatments for the millions suffering from abnormal bladder sensation and urgency.

## Conclusions

The bladder trigone stands out as a highly specialized and dynamically responsive sensory organ within the intricate landscape of the LUT. Its fundamental role lies in the sophisticated integration of a multitude of signals - including mechanical stretch, chemical irritants, and various peptidergic cues - all of which converge to precisely regulate the complex process of micturition. A deeper and more comprehensive understanding of the trigone's unique afferent architecture, characterized by its dense and diverse nerve endings, coupled with an elucidation of its intricate molecular signaling pathways, is paramount.

This enhanced knowledge is not merely academic; it is poised to drive the development of highly precise diagnostic tools and groundbreaking, targeted therapies for the pervasive and often debilitating spectrum of LUTS. In fact, the clinical evidence for the effectiveness of trigone-centric treatment is still limited. Nonetheless, the bladder trigone's intricate sensory mechanisms are attractive treatment targets for innovative diagnostic and therapeutic strategies. By unraveling the trigone's secrets, we are on the cusp of ushering in a transformative new era of personalized bladder medicine, offering hope for improved quality of life for countless individuals.

## References

[1] de Rijk M, Hentzen C, Selai C (2025). Systematic evaluation of lower urinary tract sensations to improve management of LUTS: ICI-RS 2024. Neurourol Urodyn.

[2] de Groat WC, Griffiths D, Yoshimura N (2015). Neural control of the lower urinary tract. Compr Physiol.

[3] Andersson KE (2002). Bladder activation: afferent mechanisms. Urology.

[4] Andersson KE, Arner A (2004). Urinary bladder contraction and relaxation: physiology and pathophysiology. Physiol Rev.

[5] Tanagho EA, Meyers FH, Smith DR (1968). The trigone: anatomical and physiological considerations. I. In relation to the ureterovesical junction. J Urol.

[6] Dixon JS, Gilpin CJ (1987). Presumptive sensory axons of the human urinary bladder: a fine structural study. J Anat.

[7] Purves JT, Spruill L, Rovner E (2017). A three dimensional nerve map of human bladder trigone. Neurourol Urodyn.

[8] Forrest SL, Osborne PB, Keast JR (2013). Characterization of bladder sensory neurons in the context of myelination, receptors for pain modulators, and acute responses to bladder inflammation. Front Neurosci.

[9] Viana R, Batourina E, Huang H (2007). The development of the bladder trigone, the center of the anti-reflux mechanism. Development.

[10] Paner GP, Ro JY, Wojcik EM, Venkataraman G, Datta MW, Amin MB (2007). Further characterization of the muscle layers and lamina propria of the urinary bladder by systematic histologic mapping: implications for pathologic staging of invasive urothelial carcinoma. Am J Surg Pathol.

[11] DeLancey J, Gosling J, Creed K (2002). Gross anatomy and cell biology of the lower urinary tract. Second International Consultation on Incontinence.

[12] Jost SP, Gosling JA, Dixon JS (1989). The morphology of normal human bladder urothelium. J Anat.

[13] Jafari NV, Rohn JL (2022). The urothelium: a multi-faceted barrier against a harsh environment. Mucosal Immunol.

[14] Acharya P, Beckel J, Ruiz WG, Wang E, Rojas R, Birder L, Apodaca G (2004). Distribution of the tight junction proteins ZO-1, occludin, and claudin-4, -8, and -12 in bladder epithelium. Am J Physiol Renal Physiol.

[15] Wakabayashi Y, Tomoyoshi T, Fujimiya M, Arai R, Maeda T (1993). Substance P-containing axon terminals in the mucosa of the human urinary bladder: pre-embedding immunohistochemistry using cryostat sections for electron microscopy. Histochemistry.

[16] Smet PJ, Moore KH, Jonavicius J (1997). Distribution and colocalization of calcitonin gene-related peptide, tachykinins, and vasoactive intestinal peptide in normal and idiopathic unstable human urinary bladder. Lab Invest.

[17] Wiseman OJ, Brady CM, Hussain IF (2002). The ultrastructure of bladder lamina propria nerves in healthy subjects and patients with detrusor hyperreflexia. J Urol.

[18] DeLancey J, Gosling J, Creed K (2002). Urethral lamina propria. Second International Consultation on Incontinence.

[19] Andersson KE, McCloskey KD (2014). Lamina propria: the functional center of the bladder?. Neurourol Urodyn.

[20] de Rijk MM, Peter S, Wolf-Johnston A, Heesakkers J, van Koeveringe GA, Birder LA (2024). Quantification of aging-related decreases in sensory innervation of the bladder trigone in rats. Int Neurourol J.

[21] Marshall KL, Saade D, Ghitani N (2020). PIEZO2 in sensory neurons and urothelial cells coordinates urination. Nature.

[22] Thor KB, de Groat WC (2010). Neural control of the female urethral and anal rhabdosphincters and pelvic floor muscles. Am J Physiol Regul Integr Comp Physiol.

[23] Birder L, Andersson KE (2013). Urothelial signaling. Physiol Rev.

[24] Brierley SM, Goh KG, Sullivan MJ, Moore KH, Ulett GC, Grundy L (2020). Innate immune response to bacterial urinary tract infection sensitises high-threshold bladder afferents and recruits silent nociceptors. Pain.

[25] Lepiarczyk E, Maździarz M, Paukszto Ł (2025). Transcriptomic characterization of the porcine urinary bladder trigone following intravesical administration of resiniferatoxin: insights from high-throughput sequencing. Toxins (Basel).

[26] Birder L, de Groat W, Mills I, Morrison J, Thor K, Drake M (2010). Neural control of the lower urinary tract: peripheral and spinal mechanisms. Neurourol Urodyn.

[27] Dalghi MG, Montalbetti N, Carattino MD, Apodaca G (2020). The urothelium: Life in a liquid environment. Physiol Rev.

[28] Grundy L, Daly DM, Chapple C, Grundy D, Chess-Williams R (2018). TRPV1 enhances the afferent response to P2X receptor activation in the mouse urinary bladder. Sci Rep.

[29] Szczot M, Liljencrantz J, Ghitani N (2018). PIEZO2 mediates injury-induced tactile pain in mice and humans. Sci Transl Med.

[30] Dixon JS, Gosling JA (1983). Histology and fine structure of the muscularis mucosae of the human urinary bladder. J Anat.

[31] Apostolidis A, Popat R, Yiangou Y (2005). Decreased sensory receptors P2X3 and TRPV1 in suburothelial nerve fibers following intradetrusor injections of botulinum toxin for human detrusor overactivity. J Urol.

[32] Brady CM, Apostolidis A, Yiangou Y (2004). P2X3-immunoreactive nerve fibres in neurogenic detrusor overactivity and the effect of intravesical resiniferatoxin. Eur Urol.

[33] Landon DN, Wiseman OJ (2001). A Pacinian corpuscle in the human bladder lamina propria. J Neurocytol.

[34] Avelino A, Cruz F, Coimbra A (1999). Intravesical resiniferatoxin desensitizes rat bladder sensory fibres without causing intense noxious excitation. A c-fos study. Eur J Pharmacol.

[35] Cockayne DA, Hamilton SG, Zhu QM (2000). Urinary bladder hyporeflexia and reduced pain-related behaviour in P2X3-deficient mice. Nature.

[36] Beckel JM, Daugherty SL, Tyagi P, Wolf-Johnston AS, Birder LA, Mitchell CH, de Groat WC (2015). Pannexin 1 channels mediate the release of ATP into the lumen of the rat urinary bladder. J Physiol.

[37] Ewerton F, Cruz F, Kapp M, Klein S, Roehm P, Chapple C (2024). Efficacy and safety of eliapixant in overactive bladder: the 12-week, randomised, placebo-controlled phase 2a OVADER study. Eur Urol Focus.

[38] Kitagawa Y, Wada M, Kanehisa T (2013). JTS-653 blocks afferent nerve firing and attenuates bladder overactivity without affecting normal voiding function. J Urol.

[39] Dalghi MG, Ruiz WG, Clayton DR (2021). Functional roles for PIEZO1 and PIEZO2 in urothelial mechanotransduction and lower urinary tract interoception. JCI Insight.

[40] Andersson KE, Behr-Roussel D, Denys P, Giuliano F (2022). Acute intravesical capsaicin for the study of TRPV1 in the lower urinary tract: clinical relevance and potential for innovation. Med Sci (Basel).

[41] Lai HH, Qiu CS, Crock LW, Morales ME, Ness TJ, Gereau RW 4th (2011). Activation of spinal extracellular signal-regulated kinases (ERK) 1/2 is associated with the development of visceral hyperalgesia of the bladder. Pain.

[42] Nandigama R, Bonitz M, Papadakis T, Schwantes U, Bschleipfer T, Kummer W (2010). Muscarinic acetylcholine receptor subtypes expressed by mouse bladder afferent neurons. Neuroscience.

[43] Yoshimura N, Oguchi T, Yokoyama H (2014). Bladder afferent hyperexcitability in bladder pain syndrome/interstitial cystitis. Int J Urol.

[44] Wada N, Shimizu T, Shimizu N (2018). The effect of neutralization of nerve growth factor (NGF) on bladder and urethral dysfunction in mice with spinal cord injury. Neurourol Urodyn.

[45] Liu HT, Tyagi P, Chancellor MB, Kuo HC (2009). Urinary nerve growth factor level is increased in patients with interstitial cystitis/bladder pain syndrome and decreased in responders to treatment. BJU Int.

[46] Igarashi T, Tyagi P, Mizoguchi S (2021). Therapeutic effects of nerve growth factor-targeting therapy on bladder overactivity in rats with prostatic inflammation. Prostate.

[47] Liu L, Mansfield KJ, Kristiana I, Vaux KJ, Millard RJ, Burcher E (2007). The molecular basis of urgency: regional difference of vanilloid receptor expression in the human urinary bladder. Neurourol Urodyn.

[48] Sánchez Freire V, Burkhard FC, Schmitz A, Kessler TM, Monastyrskaya K (2011). Structural differences between the bladder dome and trigone revealed by mRNA expression analysis of cold-cut biopsies. BJU Int.

[49] Komesu YM, Ketai LH, Mayer AR, Teshiba TM, Rogers RG (2011). Functional MRI of the brain in women with overactive bladder: brain activation during urinary urgency. Female Pelvic Med Reconstr Surg.

[50] Akiyama Y, Maeda D, Morikawa T (2018). Digital quantitative analysis of mast cell infiltration in interstitial cystitis. Neurourol Urodyn.

[51] Mukerji G, Yiangou Y, Agarwal SK, Anand P (2006). Transient receptor potential vanilloid receptor subtype 1 in painful bladder syndrome and its correlation with pain. J Urol.

[52] Moldwin RM, Nursey V, Yaskiv O (2022). Immune cell profiles of patients with interstitial cystitis/bladder pain syndrome. J Transl Med.

[53] Roberts MW, Sui G, Wu R (2020). TRPV4 receptor as a functional sensory molecule in bladder urothelium: stretch-independent, tissue-specific actions and pathological implications. FASEB J.

[54] Salazar BH, Hoffman KA, Zhang C, Zhang Y, Cruz Y, Boone TB, Munoz A (2019). Modulatory effects of intravesical P2X2/3 purinergic receptor inhibition on lower urinary tract electromyographic properties and voiding function of female rats with moderate or severe spinal cord injury. BJU Int.

[55] Salazar BH, Hoffman KA, Zhang C, Kavanagh A, Zhang Y, Boone TB, Munoz A (2017). Electrical activity of the bladder is attenuated by intravesical inhibition of P2X2/3 receptors during micturition in female rats. Int Neurourol J.

[56] Apostolidis A, Gonzales GE, Fowler CJ (2006). Effect of intravesical resiniferatoxin (RTX) on lower urinary tract symptoms, urodynamic parameters, and quality of life of patients with urodynamic increased bladder sensation. Eur Urol.

[57] Rios LA, Panhoca R, Mattos D Jr, Srugi M, Bruschini H (2007). Intravesical resiniferatoxin for the treatment of women with idiopathic detrusor overactivity and urgency incontinence: a single dose, 4 weeks, double-blind, randomized, placebo controlled trial. Neurourol Urodyn.

[58] Koivisto AP, Belvisi MG, Gaudet R, Szallasi A (2022). Advances in TRP channel drug discovery: from target validation to clinical studies. Nat Rev Drug Discov.

[59] Montalbetti N, Dalghi MG, Parakala-Jain T, Clayton D, Apodaca G, Carattino MD (2023). Antinociceptive effect of the calcitonin gene-related peptide receptor antagonist BIBN4096BS in mice with bacterial cystitis. Am J Physiol Renal Physiol.

[60] (2025). Migraine medication effects on urinary symptoms. https://clinicaltrials.gov/study/NCT06212661.

[61] Jiang YH, Yu WR, Kuo HC (2020). Therapeutic effect of botulinum toxin A on sensory bladder disorders-from bench to bedside. Toxins (Basel).

[62] Manecksha RP, Cullen IM, Ahmad S (2012). Prospective randomised controlled trial comparing trigone-sparing versus trigone-including intradetrusor injection of abobotulinumtoxin A for refractory idiopathic detrusor overactivity. Eur Urol.

[63] El-Hefnawy AS, Elbaset MA, Taha DE, Wadie BS, Kenawy M, Shokeir AA, Badry ME (2021). Trigonal-sparing versus trigonal-involved Botox injection for treatment of idiopathic overactive bladder: a randomized clinical trial. Low Urin Tract Symptoms.

[64] Peters KM, Carrico DJ, Perez-Marrero RA, Khan AU, Wooldridge LS, Davis GL, Macdiarmid SA (2010). Randomized trial of percutaneous tibial nerve stimulation versus Sham efficacy in the treatment of overactive bladder syndrome: results from the SUmiT trial. J Urol.

[65] Staskin DR, Peters KM, MacDiarmid S, Shore N, de Groat WC (2012). Percutaneous tibial nerve stimulation: a clinically and cost effective addition to the overactive bladder algorithm of care. Curr Urol Rep.

[66] Siegel S, Noblett K, Mangel J (2015). Results of a prospective, randomized, multicenter study evaluating sacral neuromodulation with InterStim therapy compared to standard medical therapy at 6-months in subjects with mild symptoms of overactive bladder. Neurourol Urodyn.

[67] Yune JJ, Shen JK, Pierce MA, Hardesty JS, Kim J, Siddighi S (2018). Intravesical electrical stimulation treatment for overactive bladder: an observational study. Investig Clin Urol.

[68] Chew LE, Hannick JH, Woo LL, Weaver JK, Damaser MS (2025). The future of urodynamics: innovations, challenges, and possibilities. Neurourol Urodyn.

[69] Tsiapakidou S, Apostolidis A, Pantazis K, Grimbizis GF, Mikos T (2021). The use of urinary biomarkers in the diagnosis of overactive bladder in female patients. A systematic review and meta-analysis. Int Urogynecol J.

[70] Santo B, Fink EE, Krylova AE (2025). Exploring the utility of snRNA-seq in profiling human bladder tissue: a comprehensive comparison with scRNA-seq. iScience.

